# Foliar application of selenium increased selenium accumulation, speciation, and bioaccessibility, as well as the yield and nutritional quality of sweet maize

**DOI:** 10.3389/fpls.2025.1733890

**Published:** 2025-12-19

**Authors:** Emmanuel Osei Asamoah, Solomon Musoke Ssemalawa, Ofori Prince Danso, Yuanqi Wang, Muhammad Raza Farooq, Pincheng Rao, Haoyuan Sun, Yukun Guo, Xuebin Yin, Youtao Chen

**Affiliations:** 1 College of Agriculture, Anhui Science and Technology University, Chuzhou, China; 2 School of Earth and Space Sciences, University of Science and Technology of China, Hefei, Anhui, China; 3 College of Agriculture, Shanxi Agricultural University, Taiyuan, China; 4 School of Plant Protection, Anhui Agricultural University, Hefei, China; 5 Institute of Functional Agriculture (Food) Science and Technology at Yangtze River Delta, Anhui Science and Technology University, Chuzhou, China; 6 Anhui Province Key Laboratory of Functional Agriculture and Functional Food, Anhui Science and Technology University, Chuzhou, China

**Keywords:** foliar application, selenite, sweet maize, yield, antioxidant, mineral elements, speciation, bioaccessibility

## Abstract

**Introduction:**

Selenium (Se) deficiency remains a significant global nutritional issue, emphasizing the need for efficient crop-based biofortification interventions.

**Methods:**

This study examined the mechanistic responses of sweet maize (*Zea mays L*.) to foliar Se fertilization (0, 20, 40, and 60 g ha^−1^), focusing on antioxidant regulation, physiological traits, nutrient metabolism, Se speciation, and Se bioaccessibility.

**Results:**

Moderate Se doses (20–40 g ha^−1^) enhanced chlorophyll retention and photosynthetic efficiency, accompanied by increased activities of superoxide dismutase (SOD), peroxidase (POD), and catalase (CAT), as well as 22.8% reduction in malondialdehyde (MDA), indicating improved redox homeostasis. These biochemical improvements facilitated higher assimilate accumulation, resulting in a 2–7% increase in fresh cob yield and enhanced levels of soluble sugars, amylose, protein, vitamin C, and key micronutrients (magnesium, iron, copper, manganese). However, excessive Se (60 g ha^−1^) caused oxidative imbalance, leading to decreased enzyme activity and reduced yield. Kernel Se concentration increased significantly with Se supply, dominated by selenomethionine (SeMet) (82.3% of total Se), exhibiting high *in vitro* bioaccessibility (35.6% gastric, 76.0% intestinal).

**Discussion:**

The coordinated regulation of antioxidant defense and nutrient metabolism under optimal Se supply enhances both plant physiological performance and the nutritional bioefficacy of edible kernels, providing a mechanistic framework for sustainable Se biofortification.

## 1 Introduction

Addressing the persistent challenge of “hidden hunger” and micronutrient deficiencies in a growing global population requires strategies that enhance both crop yield and nutritional quality (Yilmaz and Yilmaz, 2025).

Micronutrient malnutrition affects more than two billion people, particularly in areas where diets are predominantly composed of staple cereals (Ashraf, 2025). Agronomic interventions that increase nutrient density and crop productivity provide a sustainable pathway to improve dietary quality and strengthen food system resilience (Akpojevwe Abafe et al., 2025). Maize (*Zea mays L.*) is a major staple crop that contributes substantially to global caloric and nutrient intake (Shio et al., 2022; Shah et al., 2025). Among its subspecies, sweet maize (*Zea mays ssp. saccharata*) is notable for its high concentrations of soluble sugars, vitamins, and essential minerals (Swapna et al., 2020), offering both nutritional and sensory value. Its short growth cycle, active carbohydrate metabolism, and broad dietary acceptance make sweet maize an ideal model for nutrient biofortification research (Sidahmed et al., 2025). However, despite its growing consumption and economic acceptance, studies on selenium (Se) enrichment, speciation, and bioaccessibility in sweet maize remain limited.

Biofortification enhances the micronutrient content of crops during growth, thereby increasing dietary mineral intake without the need for post-harvest fortification (Malézieux et al., 2024; Oztekin and Buyuktuncer, 2025). Among the target micronutrients, Se is essential for human health. It is a key component of selenoproteins that regulate antioxidant defense, thyroid hormone metabolism, and immune regulation (Shahidin et al., 2025). More than one billion people worldwide are affected by Se deficiency, particularly in regions with Se-poor soils (Danso et al., 2023). Selenium deficiency is associated with impaired immunity, cardiomyopathy, and thyroid dysfunction (Wang P. et al., 2023). Utilizing staple crops as vehicles for Se delivery offers a cost-effective, population-scale intervention with substantial public health benefits (Gorni et al., 2025; Li J. et al., 2025). From an agronomic perspective, foliar Se fertilization is one of the most efficient methods for biofortification (Galić et al., 2021). Unlike soil Se application, foliar spraying bypasses Se immobilization, allows precise control of dose and timing, and facilitates effective translocation to edible tissues (Ikram et al., 2024; Gorni et al., 2025; Li H. et al., 2025). Once absorbed, Se is incorporated into selenoenzymes such as glutathione peroxidase and thioredoxin reductase, which regulate redox homeostasis, limit reactive oxygen species accumulation, and stabilize photosynthetic membranes (Li et al., 2022; Li J. et al., 2025). These biochemical effects can improve photosynthetic efficiency, enhance nutrient assimilation, and increase yield stability under stress conditions (Danso et al., 2023).

Selenium shares key metabolic pathways with sulfur (S), particularly in their uptake and assimilation routes (Gui et al., 2022), and also interacts functionally with mineral elements such as iron (Fe), zinc (Zn), manganese (Mn), and copper (Cu) through their roles in amino acid biosynthesis and antioxidant enzyme activity (Gui et al., 2022; Deng et al., 2025). A moderate Se supply can optimize nutrient homeostasis and metabolic efficiency, while excessive Se disrupts these pathways, leading to oxidative imbalance and nutrient antagonism (Dou et al., 2021). This biphasic dose–response, known as Se hormesis, highlights the need for precise dose management to maximize nutritional benefits without causing phytotoxicity (Gupta and Gupta, 2017; Danso et al., 2023; Li J. et al., 2025). The nutritional efficacy of Se-enriched crops depends not only on total Se accumulation but also on Se speciation and bioaccessibility (An et al., 2025; Farooq et al., 2025). Organic Se forms, particularly selenomethionine (SeMet), selenocysteine (SeCys), and methylselenocysteine (MeSeCys), are more bioavailable and offer greater physiological benefits compared to inorganic species (Abdalla et al., 2025; Farooq et al., 2025). The distribution of these species is affected by crop genotype, metabolic status, environmental conditions, and fertilization strategies. Despite the potential of sweet maize as a functional food with high nutritional value, Se speciation and bioaccessibility in this crop remain poorly characterized (Li H. et al., 2025). Integrating field agronomy with biochemical and nutritional analyses can clarify how foliar Se application affects Se assimilation, transformation, and transfer to kernels. Moreover, Se biofortification may enhance the composition of essential nutrients, improving the overall nutritional quality of sweet maize (Gui et al., 2022). Previous research on grain and waxy maize has shown that optimal Se levels can improve protein synthesis, carbohydrate metabolism, vitamin C concentration, and micronutrient accumulation (Naseem et al., 2021; Lu et al., 2024). These findings suggest a coordinated metabolic response between Se-mediated redox regulation and nutrient biosynthesis. Understanding these interconnections is essential for developing biofortification strategies that enhance both yield and nutritional quality while maintaining physiological balance.

Despite growing interest in Se biofortification, the mechanistic relationships among foliar Se application, antioxidant regulation, kernel nutrient composition, Se speciation, and *in vitro* bioaccessibility under field conditions remain unclear. Furthermore, the threshold separating beneficial from toxic Se levels in sweet maize has not been established. Clarifying these relationships is crucial for developing practical, mechanistically grounded biofortification guidelines that can be effectively implemented in production systems. This study evaluated the effects of foliar Se application on various physiological traits, antioxidant enzyme activity, kernel nutritional composition, mineral element accumulation, Se concentration, Se speciation, and *in vitro* bioaccessibility in sweet maize. We hypothesized that moderate Se doses enhance growth and kernel nutritional quality by modulating redox homeostasis, nutrient assimilation, and Se–S metabolic interactions. At the same time, excessive Se disrupts these processes and induces oxidative imbalance. By integrating agronomic, physiological, and nutritional perspectives, this study seeks to establish the mechanistic basis for Se biofortification in sweet maize and identify optimal Se levels for producing nutritionally enriched, bioavailable, and health-promoting kernels.

## 2 Materials and methods

### 2.1 Description of experimental site

A field experiment was conducted from July to October 2024 at the Wangying Village Experimental Base, Chuzhou, Anhui Province, China (32°07'40"N, 118°24'23"E). The site is characterized by a subtropical humid monsoon climate, with an average daily temperature of 30 °C and total rainfall of 526.7 mm during the experimental period. The soil is classified as a haplic luvisol (clay loam). Baseline soil samples were collected from ten random points at a depth of 0–20 cm prior to land preparation. The physicochemical properties, including baseline Se concentration, are summarized in **Table 1**.

**Table 1:** Characterization of baseline physical and chemical soil properties in the experimental field.

Soil property	Value
pH	5.96
Organic matter content (g kg^−1^)	12.80
Total Nitrogen (g kg^−1^)	0.52
Total Phosphorus (g kg^−1^)	0.57
Total Potassium (g kg^−1^)	1.40
Alkaline nitrogen (mg kg^−1^)	84.04
Available phosphorus (mg kg^−1^)	45.30
Available potassium (mg kg^−1^)	167.90
Total Selenium (mg kg^-1^)	0.16

### 2.2 Experimental design and materials

The experiment used the sweet maize hybrid ‘Feng Nuo 168’ obtained from Anhui Science and Technology University. This cultivar is distinguished by its high soluble sugar content and favorable kernel development, making it well-suited for biofortification studies. The average yield is 1.27 t ha^−1^ (fresh ear basis), with a growth duration of approximately 75 days from seedling emergence to fresh ear harvest. A randomized complete block design with three replicates was employed. Each block comprised all four foliar Se treatments randomly assigned to plots. The treatment consisted of: 0 g ha^−1^ (control, CK), 20 g ha^−1^ (Se1), 40 g ha^−1^ (Se2), and 60 g ha^−1^ (Se3). Twelve plots were established, each measuring 7.5 m × 6.5 m (48.75 m^2^). Selenium was applied as sodium selenite (Na_2_SeO_3_·5H_2_O, Sigma-Aldrich) dissolved in water with 0.2% Tween-20 to enhance foliar adherence and absorption. A spray volume of 500 L ha^−1^ was applied at the tassel initiation stage using a compression sprayer, when Se uptake is most efficient for kernel enrichment. The selected doses were based on prior studies in fox tail millet showing effective Se accumulation without phytotoxicity (Chen et al., 2024). Baseline fertilization included 103 kg ha^−1^ each of N, P_2_O_5_, and K_2_O applied before sowing, followed by 84 kg ha^−1^ urea (46% N) top-dressed at the jointing stage. Seeds were sown in July 2024 at two seeds per hill, with a spacing of 60 cm between rows and 40 cm within rows. Thinning was conducted 15 days after emergence, leaving one plant per stand, resulting in a final plant density of 41,667 plants ha^−1^. Weed management was performed manually twice, and pest control included two applications of lambda-cyhalothrin at the jointing and small bell stages. All management practices were applied uniformly across all treatments to prevent confounding effects on Se uptake and kernel composition.

### 2.3 Sample collection, preparation, biomass, and analysis

#### 2.3.1 Agronomic trait and physiological analysis

One week before harvest, ten representative plants per plot were sampled to assess agronomic and physiological traits at the milk stage. Plant height was measured from the ground to the flag leaf using a tape measure, and internode diameter was determined at the third basal node using a digital vernier caliper. Leaf chlorophyll content was measured non-destructively on five randomly selected plants per plot using a SPAD-502 Plus meter (Konica Minolta Sensing, Inc.), with values expressed as the SPAD index. Following Shio et al (Shio et al., 2022), leaf area (cm^2^) was estimated using Equation 1:

(1) Leaf area=L×W×0.75

where L is leaf midrib length, W is leaf width, and 0.75 is a correction factor accounting for leaf shape.

Fresh ear yield, including cob and husk, was recorded by weighing harvested ears from five representative plants per plot. Additionally, these plants were partitioned into root, stem, leaf, kernel, husk, cob, and tassel. Samples were thoroughly washed, air-dried for five days, and oven-dried at 60 °C for 3 days to constant weight. Dried organs were ground to a fine powder and stored in labeled zip-lock bags for subsequent analyses.

#### 2.3.2 Antioxidant enzyme analysis

Leaf samples for antioxidant enzyme analysis were collected ten days after foliar Se application, corresponding to the period of expected peak Se incorporation into selenoenzymes. Five randomly selected plants per plot were sampled, immediately frozen in liquid nitrogen, and stored at –80 °C (Media MD-86L708 freezer) until analysis. Activities of superoxide dismutase (SOD), catalase (CAT), and peroxidase (POD) were determined spectrophotometrically using homogenized leaf extracts, following previously established protocols (Jiang et al., 2022) with minor modifications (see **Supplementary Text S11**). Malondialdehyde (MDA) concentration, as an indicator of lipid peroxidation, was measured using the thiobarbituric acid (TBA) assay according to Huang et al (Huang et al., 2020), with modifications detailed in **Supplementary Text S12**. Enzyme activities and MDA concentrations are expressed per mg of protein or g fresh weight, as indicated in the respective protocols. All assays were performed in triplicate for each plot sample to ensure reproducibility.

#### 2.3.3 Analysis of selenium content, speciation, and bioaccessibility

Total Se contents in kernel, leaf, stem, root, cob, husk, and tassel were determined using inductively coupled plasma mass spectrometry (ICP-MS, 7900, Agilent, Santa Clara, CA, USA). Samples were prepared following acid digestion protocols adapted from Zhou et al (Zhou et al., 2023), with all measurements performed in triplicate (Detailed protocols are outlined in **Supplementary Text S1**). Total Se concentrations are reported in mg kg^−1^ dry weight.

Selenium chemical speciation in kernels was analyzed using liquid chromatography coupled with ICP-MS (LC-ICP-MS) following procedures described by Zeng et al (Zeng et al., 2023) and Ma et al (Ma et al., 2022). Briefly, kernel extracts were enzymatically digested to release Se species, which were then separated and quantified as selenomethionine (SeMet), selenocysteine (SeCys), and methylselenocysteine (MeSeCys). Full methodological details, including chromatographic conditions and detection parameters, are provided in **Supplementary Text S2**.

The bioaccessible fraction of Se in kernels was assessed using a modified physiologically based extraction test (MPBET) adapted from Zeng et al (Zeng et al., 2023) and Li et al (Li et al., 2021a). This *in vitro* digestion simulates human gastrointestinal conditions to estimate the fraction of Se available for absorption. Briefly, powdered kernel samples were sequentially subjected to gastric and intestinal digestion phases, and the soluble Se fraction was quantified by ICP-MS (see **Supplementary Text S10** for detailed protocol).

#### 2.3.4 Analysis of sugar content, soluble sugars, and starch components

Soluble sugar content in kernels was determined using a UV-Visible spectrophotometer adapted from Lu et al (Lu et al., 2024). Briefly, 0.5 g of kernel flour was extracted with 5 mL of 80% ethanol, vortexed, and incubated at 85 °C for 40 min. After centrifugation, the supernatant was filtered and adjusted to 50 mL with 80% ethanol. For quantification, 1.5 mL of extract was mixed with distilled water and 0.2% anthrone reagent, incubated at 100 °C for 20 min, and absorbance was measured at 620 nm (see **Supplementary Text S3** for details).

Sugar content in fresh kernels was determined using a °Brix refractometer (Atago Co., Japan). In brief, 12g of kernels from five randomly selected ears per plot were homogenized, filtered to obtain corn milk, and applied directly to the °Brix refractometer, which is automatically calibrated and displays the Brix value corresponding to the total soluble solids content.

Total starch content was quantified using a modified anthrone colorimetry approach (Lu et al., 2024) (see **Supplementary Text S8** for full details). Apparent amylose content was determined via a dual-wavelength iodine-binding spectrophotometric method (Lu et al., 2024) (see **Supplementary Text S9**), and amylopectin content was calculated as the difference between total starch and apparent amylose.

#### 2.3.5 Analysis of crude protein, crude fat, and vitamin C content

Crude protein content was determined using the Kjeldahl method following Shio et al (Shio et al., 2022) (details are provided in **Supplementary Text S7**). Crude fat was extracted using a modified Soxhlet method (Lu et al., 2024) (see **Supplementary Text S4** for details), and vitamin C content was quantified according to Lu et al (Lu et al., 2024) with slight modifications (see **Supplementary Text S5**). All measurements are expressed per gram of dry weight.

#### 2.3.6 Analysis of macro and micro nutrient content

Concentrations of nitrogen (N), phosphorus (P), potassium (K), magnesium (Mg), manganese (Mn), iron (Fe), copper (Cu), and zinc (Zn) in kernel flour were analyzed using inductively coupled plasma optical emission spectrometry (ICP-OES) following an adapted protocol (Moisa et al., 2024) (see **Supplementary Text S6**). Prior to analysis, samples were digested using a mixture of nitric and perchloric acids, and all results are reported in mg kg^−1^ dry weight.

### 2.4 Statistical analysis

Statistical analyses were performed using one-way analysis of variance (ANOVA) in Minitab (Version 20.3; Minitab LLC) to evaluate differences among treatment means. *Post-hoc* comparisons were conducted using Tukey’s Honest Significant Difference (HSD) test, with significance defined at *p* < 0.05. All data are reported as mean ± standard deviation (SD) based on three replicates per treatment. Data visualization was done in OriginPro 2024 (OriginLab, Northampton, MA, USA).

## 3 Results

### 3.1 Sweet maize yield traits

**Figure 1:**
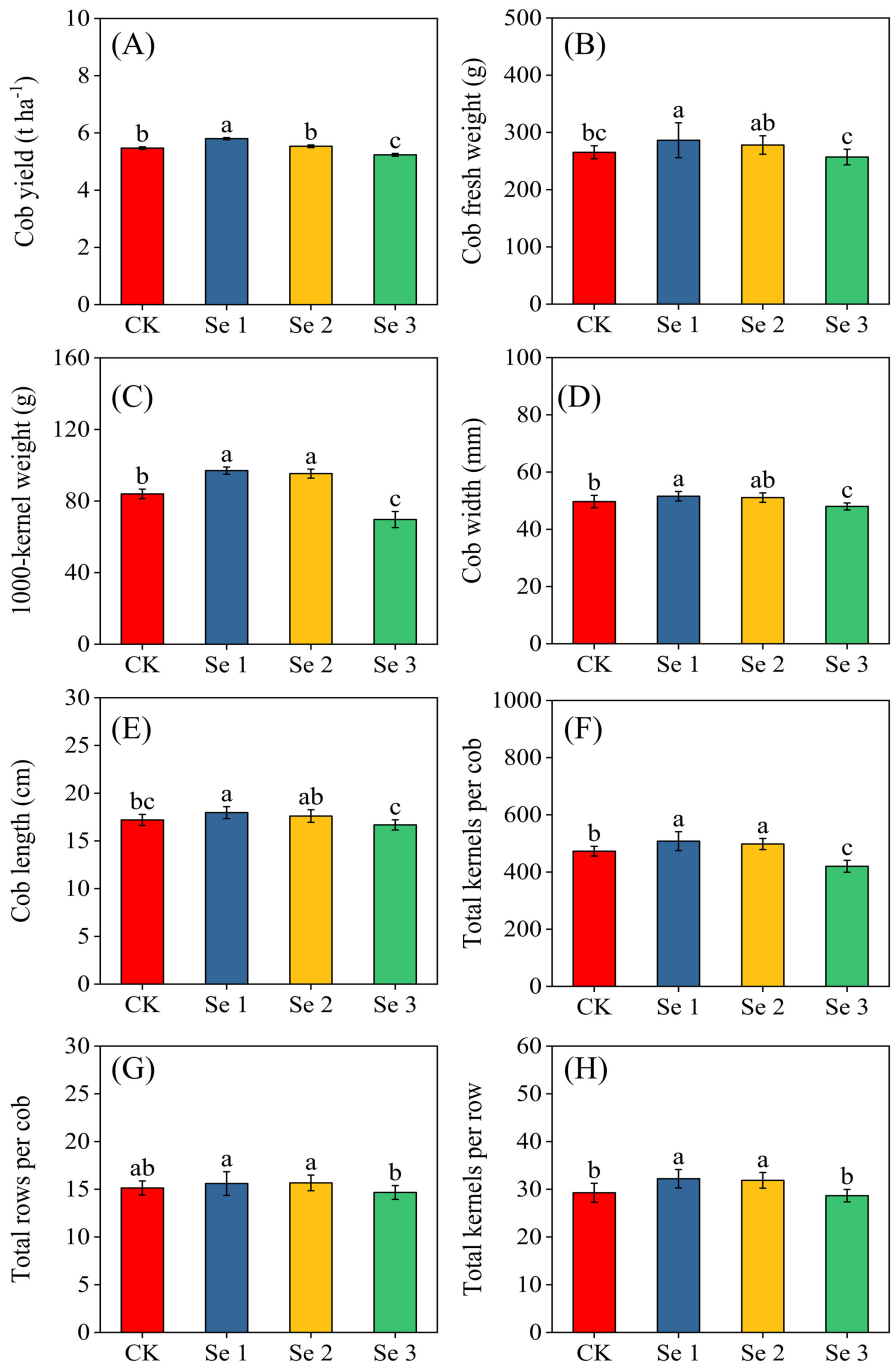
Effects of foliar application of different Se concentrations on Cob yield **(A)**; Cob fresh weight **(B)**; 1000-kernel weight **(C)**; Cob width **(D)**; Cob length **(E)**; Total kernels per cob **(F)**; Total rows per cob **(G)**; and Total kernels per row **(H)**. Bars represent means ± standard deviation (n = 10). Different letters indicate significant differences among treatments (*p* < 0.05) based on Tukey’s test. Treatments: 0 (CK), 20 g ha^−1^ (Se1), 40 g ha^−1^ (Se2), and 60 g ha^−1^ (Se3).

Foliar Se application significantly influenced several sweet maize yield components (all *p* < 0.05). Specifically, Se1 increased cob fresh yield by 5.7%, cob fresh weight by 7.3%, cob width by 3.6%, and cob length by 4.3% compared with CK (**Figures 1A–E**). Both Se1 and Se2 enhanced grain-formation traits, including 1000-kernel weight (6.1% and 5.4%), kernels per cob (6.9% and 5.1%), rows per cob (5.9% and 5.6%), and kernels per row (9.1% and 8.1%), respectively (**Figures 1C, F–H**). Although Se2 showed significant increases in ear-related traits, these improvements did not result in a significant increase in final cob yield relative to CK. In contrast, Se3 consistently reduced yield performance, reducing cob yield (5.2%), 1000-kernel weight (7.8%), cob width (3.6%), and kernels per cob (12.5%) relative to the control.

### 3.2 Biochemical and physiological analysis

**Figure 2:**
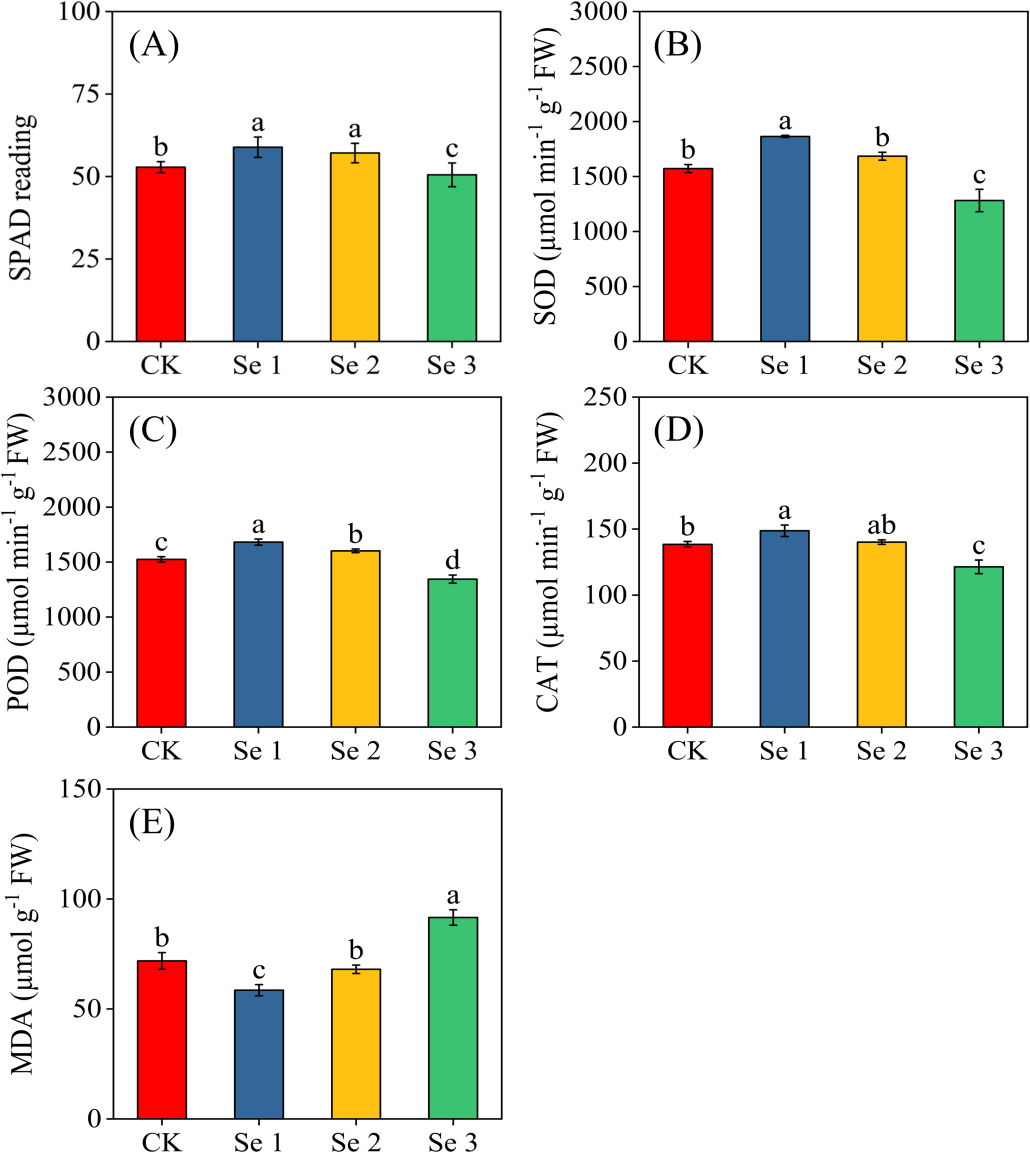
Effects of foliar application of different Se concentrations on SPAD **(A)**, SOD **(B)**, POD **(C)**, CAT **(D)**, and MDA content **(E)**. Bars represent means ± standard deviation (n = 5). Different letters indicate significant differences among treatments (*p* < 0.05) based on Tukey’s test. Treatments: 0 (CK), 20 g ha^−1^ (Se1), 40 g ha^−1^ (Se2), and 60 g ha^−1^ (Se3).

Foliar Se application significantly influenced chlorophyll content (SPAD), antioxidant enzyme activities (SOD, POD, CAT), and lipid peroxidation (MDA) in sweet maize (all *p* < 0.05; **Figures 2A–E**). Regarding chlorophyll content, moderate Se treatments enhanced SPAD values, with increases of 10.3% for Se1 and 7.5% for Se2 relative to the CK. Conversely, Se3 caused a 4.6% decrease in SPAD. In terms of antioxidant defense, Se1 significantly increased SOD by 15.7%, POD by 9.3%, and CAT by 6.9% compared with the CK. Similarly, Se2 enhanced POD activity by 4.9%, while its effects on SOD and CAT were statistically similar compared to CK. In contrast, Se3 markedly suppressed all three enzymes, reducing SOD by 22.7%, POD by 13.4%, and CAT by 14.1%. MDA levels followed similar trends, with Se1 reducing MDA content by 22.8%, indicating a decrease in oxidative stress, whereas Se3 increased MDA levels by 21.6%, reflecting elevated lipid peroxidation.

### 3.3 Agronomic traits

**Figure 3:**
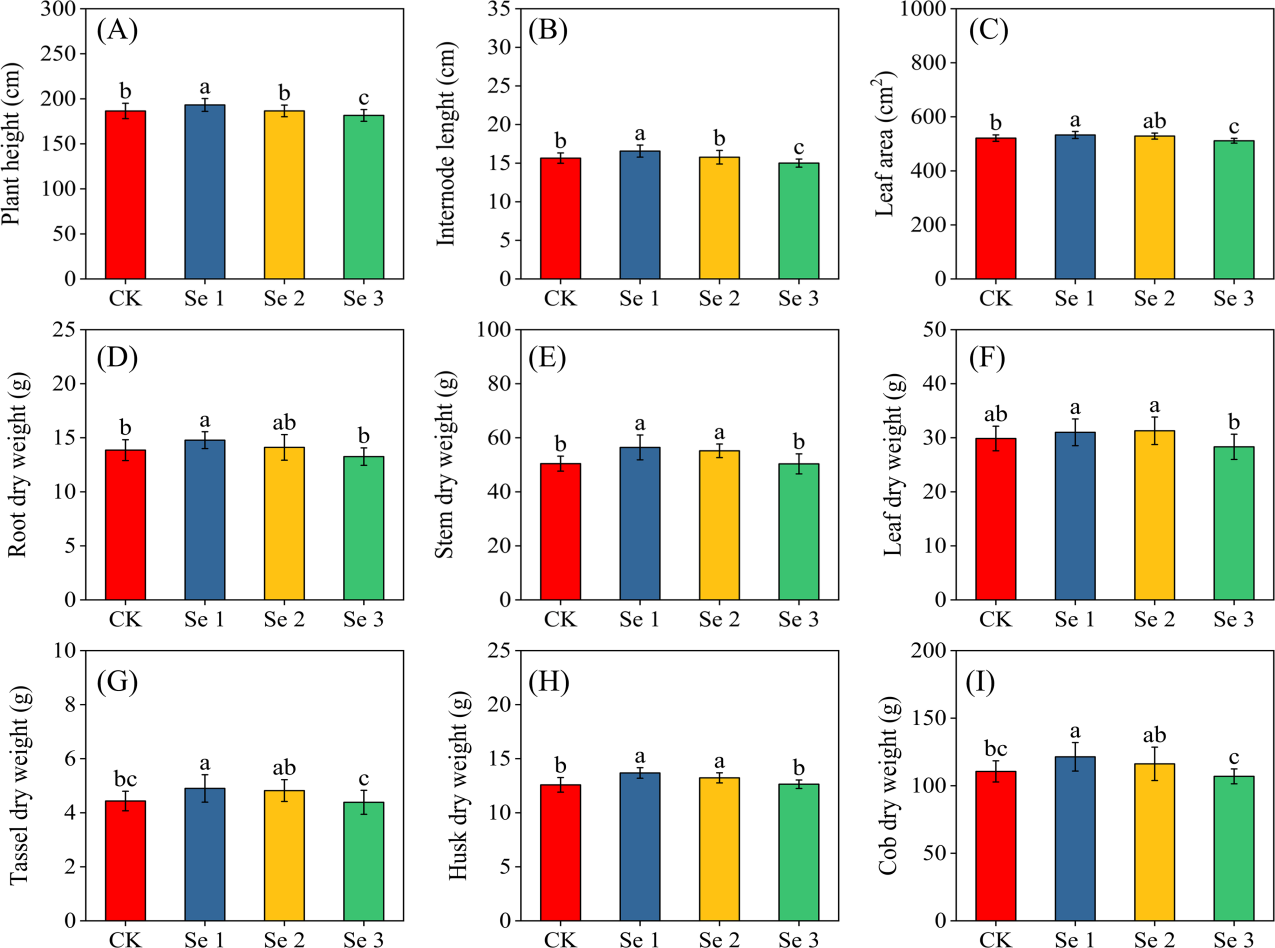
Effects of foliar application of different Se concentrations on Plant height **(A)**, Internode length **(B)**, Leaf area **(C)**, Root dry weight **(D)**; Stem dry weight **(E)**; Leaf dry weight **(F)**; Tassel dry weight **(G)**, Husk dry weight **(H)**; and Cob dry weight **(I)**; Bars represent means ± standard deviation (n = 5). Different letters indicate significant differences among treatments (*p* < 0.05) based on Tukey’s test. Treatments: 0 (CK), 20 g ha^−1^ (Se1), 40 g ha^−1^ (Se2), and 60 g ha^−1^ (Se3).

Foliar Se application significantly affected sweet maize growth and biomass accumulation (all *p* < 0.05; **Figures 3A–I**). Regarding plant height (**Figure 3A**), Se1 increased height by 3.5% compared with CK, whereas Se3 reduced it by 2.7%. Internode length (**Figure 3B**) showed a similar pattern, with Se1 increasing internode length by 5.5% and Se3 decreasing it by 4.3%. Leaf area (**Figure 3C**) was likewise enhanced under Se1 (6.6%) but suppressed under Se3 (7.4%). Biomass components followed parallel trends across treatments. Stem dry weight (**Figure 3E**) and husk dry weight (**Figure 3H**) were both higher under Se1, with CK exhibiting reductions of 10.7% and 8.0%, respectively. Cob dry weight (**Figure 3I**) and root dry weight (**Figure 3D**) were also lower in CK relative to Se1 (8.9% and 6.2%). Tassel dry weight (**Figure 3G**) showed a similar decline (9.5%). When compared with Se2, CK also recorded reduced stem (8.6%) and husk (4.9%) dry weights.

### 3.4 Nutritional quality

**Figure 4:**
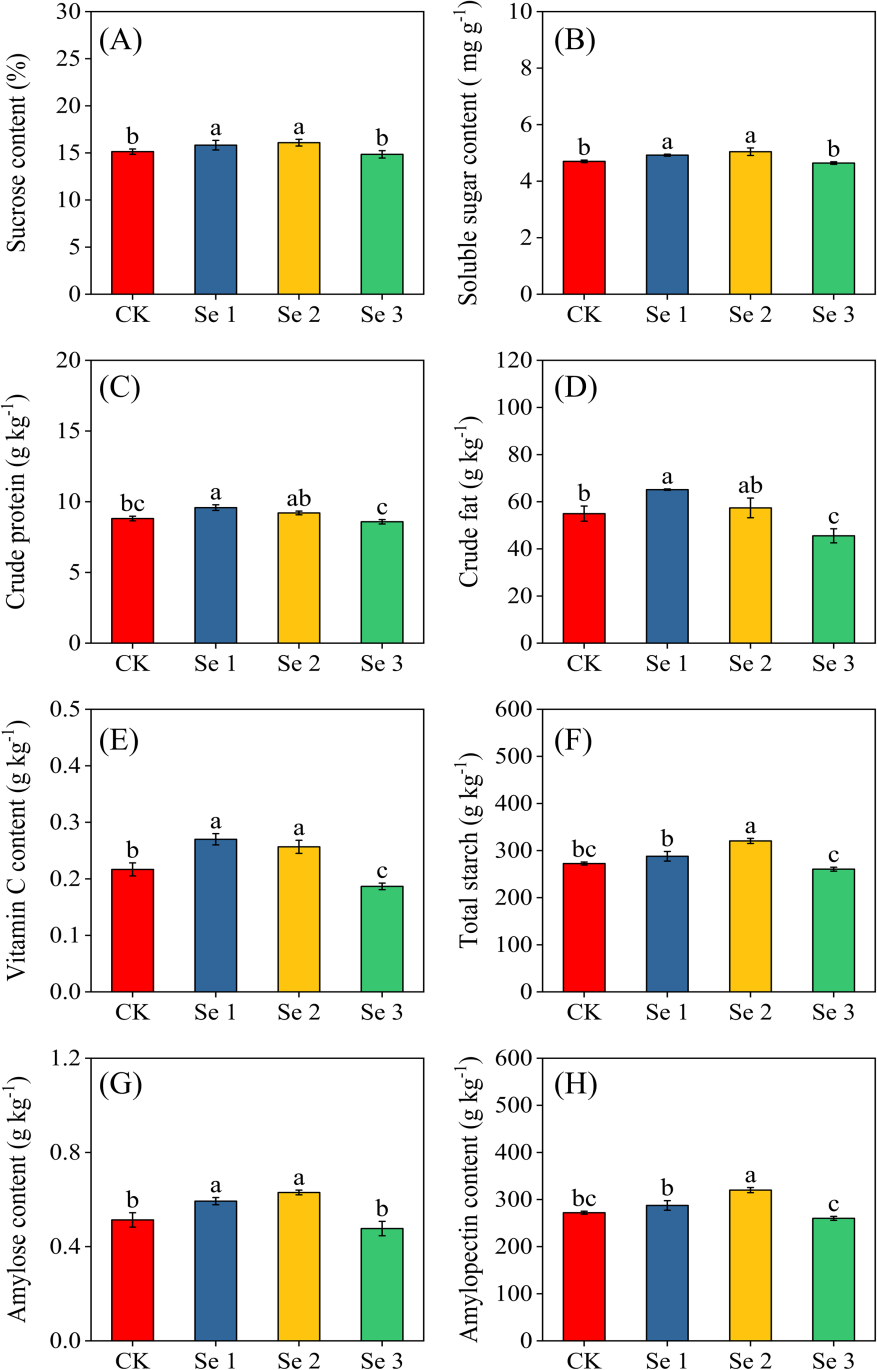
Effects of foliar application of different Se concentrations on Sugar content **(A)**; Soluble sugar content **(B)**; Crude protein **(C)**; Crude fat **(D)**; Vitamin C content **(E)**; Total starch **(F)**; Amylose **(G)**; and Amylopectin **(H)**. Bars represent means ± standard deviation (n = 5). Different letters indicate significant differences among treatments (*p* < 0.05) based on Tukey’s test. Treatments: 0 (CK), 20 g ha^−1^ (Se1), 40 g ha^−1^ (Se2), and 60 g ha^−1^ (Se3).

Foliar Se application exerted a significant and dose-dependent influence on kernel nutritional attributes, including sugar content, soluble sugar, crude protein, crude fat, vitamin C, total starch, amylose, and amylopectin (all *p* < 0.05; **Figures 4A–H**). At moderate application levels, Se1 consistently enhanced grain quality, increasing amylose (13.5%), sugar content (4.4%), soluble sugar (4.5%), vitamin C (19.8%), crude fat (15.6%), and crude protein (8.0%) relative to CK. Se1 also outperformed the high-dose treatment (Se3), showing markedly greater concentrations of amylose (19.6%), sugar content (6.5%), soluble sugar (5.7%), vitamin C (30.9%), crude fat (30.1%), and crude protein (10.4%). Se2 produced the most substantial overall improvements in kernel nutritional quality. Compared with CK, Se2 significantly increased sugar content (5.9%), total starch (14.9%), amylose (18.5%), amylopectin (15.0%), soluble sugar (6.8%), and vitamin C (15.6%). Notably, Se2 surpassed Se1 for total starch and amylopectin content, increasing by 9.1% and 10.2%, respectively. In contrast, Se3 elicited a clear decline in nutritional quality. Relative to Se2, Se3 reduced sugar content (7.7%), total starch (18.7%), amylose (24.3%), amylopectin (16.7%), soluble sugar (8.0%), and vitamin C (27.3%). Additionally, compared with CK, Se3 significantly declined vitamin C (16.1%) and crude fat (20.7%).

### 3.5 Macro and micronutrients

**Figure 5:**
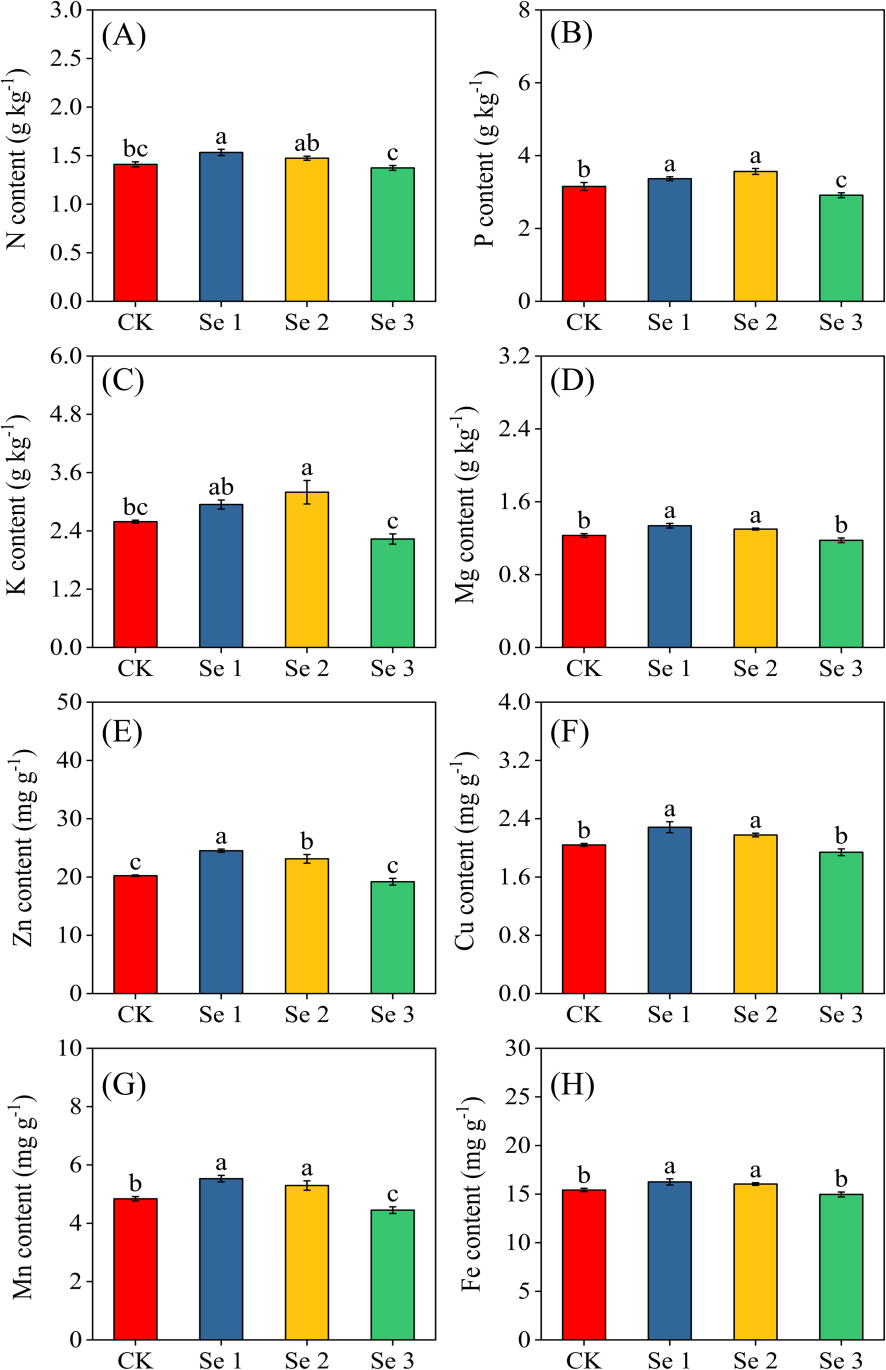
Effects of foliar application of different Se concentrations on the concentrations of macronutrients: nitrogen **(A)**, phosphorus **(B)**, potassium **(C)**, magnesium **(D)**, and micronutrients: zinc **(E)**, copper **(F)**, manganese **(G)**, and iron **(H)** in sweet maize kernels. Bars represent means ± standard deviation (n = 5). Different letters indicate significant differences among treatments (*p* < 0.05) based on Tukey’s test. Treatments: 0 (CK), 20 g ha^−1^ (Se1), 40 g ha^−1^ (Se2), and 60 g ha^−1^ (Se3).

The concentrations of kernel macronutrients (N, P, K, Mg) and micronutrients (Zn, Cu, Mn, Fe) varied significantly across Se treatments (all *p* < 0.05; **Figures 5A–H**). In terms of N content, Se1 resulted in a substantial increase of 8.0% relative to CK, while Se2 and Se3 showed no notable improvement over the control. P accumulation in kernels was enhanced by both Se1 and Se2, with increases of 6.3% and 11.6%, respectively, compared to CK. In contrast, Se3 resulted in an 8.2% reduction in P relative to the control. Regarding K content, Se2 induced a significant increase, elevating kernel K concentration by 18.9% compared to the control. For kernel Mg accumulation, both Se1 and Se2 showed elevated levels by 8.0% and 5.4%, respectively, relative to CK. Among micronutrients, Zn accumulation was highest under Se1 (17.5%), followed by Se2 (12.5%), with Se3 showing no improvement compared to CK. For Mn, Se1 resulted in the highest increase, enhancing Mn concentrations by 12.5%. Se2 also elevated Mn levels, albeit to a lesser extent (8.7%), while Se3 caused a reduction of 8.8% compared to the control. Fe content increased by 5.1% under Se1 and 3.8% under Se2, while Se3 had no significant effect on Fe levels. A similar trend was observed for Cu, with Se1 boosting Cu concentrations by 10.7%, Se2 by 6.3%, and Se3 showing no measurable effect.

### 3.6 Se content in sweet maize

**Figure 6:**
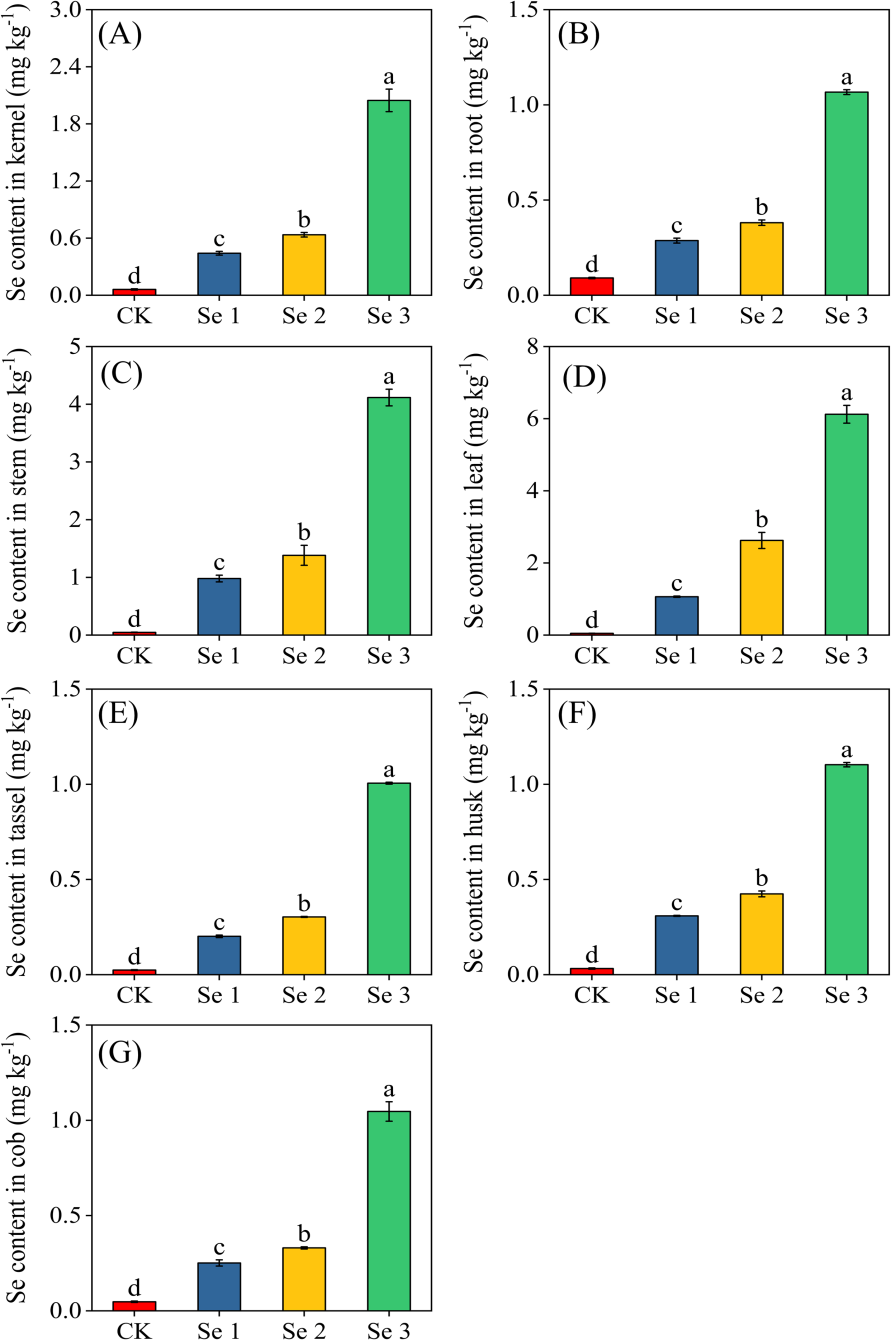
Effects of foliar application of different Se concentrations on Se content in the kernel **(A)**; root **(B)**; stem **(C)**; leaf **(D)**; tassel **(E)**; husk **(F)**; and cob **(G)**. Bars represent means ± standard deviation (n = 5). Different letters indicate significant differences among treatments (*p* < 0.05) based on Tukey’s test. Treatments: 0 (CK), 20 g ha^−1^ (Se1), 40 g ha^−1^ (Se2), and 60 g ha^−1^ (Se3).

Foliar Se application significantly increased total Se content in all sweet maize organs (*p* < 0.05) (**Figures 6A–G**). In kernels, Se1, Se2, and Se3 enhanced Se concentrations to 86.2%, 90.4%, and 97.0% above CK, respectively. Se3 further increased kernel Se content compared to Se1 and Se2 by 78.4% and 69.0%, respectively. Overall, kernel Se ranged from 0.06 mg kg^−1^ in the CK to 2.05 mg kg^−1^ in Se3. In the roots, Se content increased progressively with Se dose, reaching 68.0%, 76.2%, and 91.5% above the control under Se1, Se2, and Se3, respectively. Stems exhibited substantial accumulation at moderate to high Se doses, with Se1, Se2, and Se3 increasing Se levels by 95.2%, 96.6%, and 98.9%, respectively. For the cob, Se1 and Se2 enhanced Se content by 81.0% and 85.6%, respectively, while Se3 resulted in an even greater increase, elevating Se levels by 95.5%. Leaves displayed the highest Se enrichment, with concentrations increasing by 95.5%, 98.2%, and 99.2% under Se1, Se2, and Se3. Similarly, husks and tassels responded positively to Se application, with Se1, Se2, and Se3 increasing Se content in husks by 89.5%, 92.4%, and 97.1%, and in tassels by 87.9%, 92.0%, and 97.6%, respectively. Overall, the Se distribution within the plant showed a consistent pattern, with the highest accumulation in leaves, followed by stem, husk, tassel, kernel, cob, and root.

### 3.7 Se speciation in kernels

**Figure 7:**
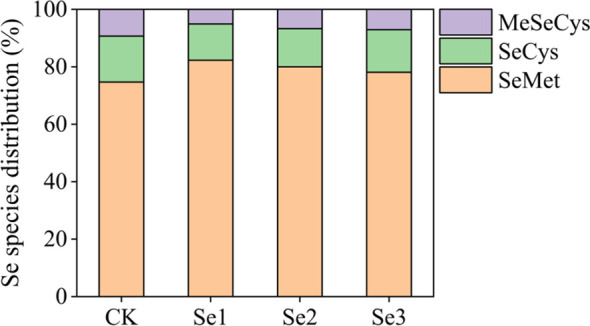
Selenium speciation in sweet maize kernels under different selenium treatments. Treatments include CK (0), Se1 (20 g ha^−1^), Se2 (40 g ha^−1^), and Se3 (60 g ha^−1^). The selenium species analyzed were selenocysteine (SeCys), methylselenocysteine (MeSeCys), and selenomethionine (SeMet). Data show the distribution and relative concentrations of each selenium species across the treatments.

Foliar Se application significantly altered the distribution of Se species in sweet maize kernels (*p* < 0.05) (**Figure 7**). In the CK group, SeMet was the dominant species, representing 74.7% of total Se, followed by SeCys (16.0%) and MeSeCys (9.3%). Across all Se treatments, SeMet remained the predominant form, but its relative proportion differed significantly among doses. Se1 increased SeMet to 82.3%, a significant rise compared with CK, while SeCys and MeSeCys declined to 12.6% and 5.1%, respectively. Se2 also elevated SeMet (80.0%), but to a lesser extent than Se1, with SeCys reaching 13.3% and MeSeCys increasing slightly to 6.7%. Under Se3, SeMet accounted for 78.1% of total Se, accompanied by moderate increases in SeCys (14.8%) and MeSeCys (7.1%), relative to CK.

### 3.8 Se bioaccessibility

**Table 2** presents the total Se content and the estimated bioaccessibility in the gastric (G) and gastrointestinal (GI) fractions of sweet maize kernels, calculated using Eq. (2) (see **Supplementary Text S10**). In the G fraction, bioaccessibility ranged from 14.67% in the CK to 35.60% under Se3. Specifically, Se1 exhibited 23.67%, Se2 30.33%, and Se3 35.60%, indicating a progressive increase with higher Se application rates. Similarly, the GI fraction showed consistently higher bioaccessibility compared to the G fraction, ranging from 37.67% in the CK to 76.00% under Se3. Treatments followed the same ascending trend, with Se1 at 51.33%, Se2 at 61.00%, and Se3 achieving the highest bioaccessibility at 76.00%.

**Table 2:** Total Se content, Bioaccessible Se content, and Se bioaccessibility in gastric (G) and gastrointestinal (GI) fractions of sweet maize kernels.

Treatment	Total Se content (mg/kg)	G fraction		GI fraction	
		Bioaccessible Se (mg/kg)	Bioaccessibility (%)	Bioaccessible Se (mg/kg)	Bioaccessibility (%)
CK	0.061 ± 0.001	0.009 ± 0.002	14.67 ± 3.51^c^	0.020 ± 0.002	37.67 ± 2.52^d^
Se1	0.441 ± 0.020	0.104 ± 0.014	23.67 ± 3.51^b^	0.173 ± 0.018	51.33 ± 3.51^c^
Se2	0.636 ± 0.024	0.193 ± 0.009	30.33 ± 1.53^a^	0.270 ± 0.018	61.00 ± 3.61^b^
Se3	2.047 ± 0.118	0.768 ± 0.065	37.67 ± 4.73^a^	0.967 ± 0.075	76.00 ± 4.58^a^

Bioaccessibility (%) is expressed as the mean ± SE (n = 3). For each digestion phase (gastric or intestinal), means followed by different lowercase letters indicate significant differences among treatments according to one-way ANOVA followed by Tukey’s HSD test (α = 0.05).

## 4 Discussion

### 4.1 Effect of foliar selenium application on sweet maize plant physiological, biochemical, and yield processes

Although Se is not considered essential for higher plants, its regulatory role in modulating redox biochemistry, stress signaling, and metabolic activity is increasingly recognized as influential for growth and productivity (Gao et al., 2024; Jia et al., 2025). In this study, foliar application of selenite at the tassel initiation stage elicited clear dose-dependent effects on physiological, biochemical, and yield-related traits of sweet maize. Moderate Se doses (20 and 40 g ha^−1^) enhanced chlorophyll content, photosynthetic capacity, and leaf expansion relative to the control. SPAD values increased by 10.3% under Se1 and by 7.5% under Se2, indicating that moderate Se availability supported chlorophyll stability and photosynthetic efficiency (Ikram et al., 2024). These results are consistent with earlier findings reporting moderate increases in chlorophyll content under Se fertilization in maize (Wang et al., 2022). The observed enhancement in SPAD values reflects Se reduction via glutathione-dependent pathways, promoting thiol-redox cycling that protects chlorophyll and photosystem II proteins from oxidative damage (Sun et al., 2020). By stabilizing the chloroplast redox environment, moderate Se likely facilitated more efficient electron transport and sustained carbon assimilation (Ikram et al., 2024). However, the SPAD response was not linear across the application gradient. At the highest rate (60 g ha^−1^), both chlorophyll content and leaf area declined. This pattern reflects a typical hormetic response, where moderate Se enhances chloroplast stability, but excessive Se intensifies glutathione consumption and disrupts cellular redox homeostasis (Liu et al., 2022). Under such conditions, the accumulation of selenodiglutathione and related intermediates can elevate reactive oxygen species (ROS) generation, impair pigment stability, and interfere with core components of the photosynthetic apparatus (Lv et al., 2021; Skrypnik et al., 2022). Similar threshold-dependent responses have been documented in previous studies (Naseem et al., 2021; Wang et al., 2022), reinforcing that the beneficial and inhibitory effects of Se depend on maintaining a balanced redox environment within plant tissues.

Antioxidant enzymes, including SOD, POD, and CAT, play central roles in mitigating oxidative stress by detoxifying reactive oxygen species (ROS) (Wang M. et al., 2023). In the present study, the biochemical effects of foliar selenite application were particularly evident in antioxidant metabolism. Moderate Se application significantly activated SOD, POD, and CAT while reducing MDA by 22.8% relative to the control, demonstrating improved protection against ROS-induced membrane damage. These responses reflect Se’s biochemical fate, where its intracellular reduction drives glutathione turnover, enhances thiol-redox buffering, upregulates antioxidant enzymes, and facilitates detoxification of superoxide and hydrogen peroxide (Liu et al., 2022). In contrast, Se3 suppressed antioxidant activity and increased MDA accumulation by 21.6%, indicating that high Se loads overwhelmed the glutathione pool, weakened redox homeostasis, and generated a pro-oxidative cellular environment (Bandehagh et al., 2023). These findings align with prior reports showing that supra-optimal Se disrupts redox balance, induces oxidative stress, and impairs metabolic processes essential for photosynthesis and growth (Gupta and Gupta, 2017; Dou et al., 2021). Thus, the biphasic pattern observed here reflects the dual redox-modulating function of Se: protective at low concentrations but disruptive when its detoxification capacity is exceeded (Gupta and Gupta, 2017).

These biochemical and physiological enhancements observed in this study translated into measurable yield differences. Both Se1 and Se2 increased fresh cob yield by 5.7% and 2.0%, respectively, while Se3 reduced yield by 5.2%. This pattern aligns with previous studies demonstrating Se-induced yield improvement in maize (Wang et al., 2022; Gorni et al., 2025). The enhanced yield at Se1 and Se2 is likely due to the combined effects of improved antioxidant defense, increased chlorophyll retention, and sustained photosynthetic activity during reproductive development (Wang et al., 2022). Given that kernel set and filling are particularly sensitive to oxidative stress, Se-mediated improvements in redox balance and membrane stability likely supported better pollen viability, increased silk receptivity, and more efficient assimilate partitioning to developing kernels. This interpretation is supported by the yield component responses: under Se1, cob length and total kernels per cob increased by 4.3% and 6.9%, respectively, with a modest increase in cob width, kernels per row, and 1,000-kernel weight, similar to findings by Nawaz et al (Nawaz et al., 2016) and Li et al (Li et al., 2021b). The decline in yield components at Se3 underscores the existence of an optimal Se application range, beyond which toxic effects reduce productivity (Wang et al., 2022). These improvements in yield under moderate Se application can be attributed to the reduction of oxidative stress through enhanced antioxidant activity, which helps preserve chloroplast structure and enzyme function critical for carbon assimilation (Dou et al., 2021). High SPAD values further indicate improved chlorophyll stability and delayed senescence, potentially extending photosynthetic activity during the kernel-filling period (D’Amato et al., 2020). Although the study did not directly assess reproductive traits such as pollen viability or assimilate remobilization, previous research suggests that Se may indirectly influence these processes, contributing to efficient kernel development (Marques et al., 2021; Cheng et al., 2024). Additionally, Se-induced osmotic adjustment, through the accumulation of compatible solutes accumulation (Zou et al., 2021) may have helped maintain cell turgor and physiological activity during kernel development.

### 4.2 Effect of foliar selenium application on nutritional quality and trace elements content in sweet maize kernels

The application of Se through foliar fertilization can substantially influence nutrient uptake and trace element dynamics in crops by modulating redox homeostasis, membrane stability, and the activity of metal transporters (Ikram et al., 2024). However, these responses are strongly dose-dependent, as Se serves as a beneficial micronutrient at low concentrations but can become antagonistic or toxic when exceeding physiological thresholds (Delaqua et al., 2021; Ikram et al., 2024). In this study, Se1 and Se2 notably enhanced the nutritional composition of sweet maize kernels compared to the control (**Figures 4A–H**). Under Se1, soluble sugars, crude fat, amylose, sugar content, vitamin C, and crude protein increased by 4.5%, 15.6%, 8.1%, 13.5%, 4.4%, 19.8%, and 8.0%, respectively. Se2 produced similar, though slightly lower, enhancement, while Se3 caused declines in vitamin C (16.1%) and crude fat (20.7%) relative to the control. These results align with previous studies reporting Se-induced improvements in kernel biochemical quality (Shio et al., 2022; Lu et al., 2024; Xia et al., 2024) and enhanced vitamin C content in leafy crops (Cheng et al., 2024). For instance, Lu et al (Lu et al., 2024) observed elevated starch, protein, and soluble sugars in waxy maize following foliar Se supplementation. The observed improvements in kernel nutritional quality under moderate Se supply likely reflect Se-mediated optimization of photosynthetic and metabolic activity. Previous studies have shown that optimal Se levels can sustain chlorophyll integrity, enhance acid invertase activity, and improve electron transport efficiency, thereby supporting greater carbon assimilation and assimilate partitioning to developing kernels (Wu et al., 2024; Xu et al., 2024). Se-induced enhancement of antioxidant defense capacity may further protect chloroplast membranes from oxidative damage, maintaining carbon fixation efficiency under field conditions (Kaur et al., 2018; Xu et al., 2024). Together, these processes likely contribute to sustained carbohydrate biosynthesis and starch accumulation in reproductive tissues (Lin et al., 2024).

Beyond carbohydrate metabolism, Se can influence amino acid and protein synthesis through its incorporation into seleno-amino acids and selenoproteins, which are critical for redox regulation and metabolic balance (Islam et al., 2020; Liang et al., 2020). The observed 8.0% increase in kernel crude protein under Se1 is consistent with this proposed mechanism. Conversely, Se3 appeared to disrupt nutrient metabolism, likely through redox imbalance and interference with sulfur assimilation pathways, thereby reducing protein synthesis and overall nutritional quality (Gupta and Gupta, 2017; Ikram et al., 2024). Moderate Se supplementation also enhanced the accumulation of key micronutrients in sweet maize kernels. Compared to the control, Se1 and Se2 increased iron by 5.1% and 3.8%, zinc by 17.5% and 12.5%, manganese by 12.5% and 8.7%, and Cu by 10.7% and 6.3%, respectively. In contrast, Se3 reduced phosphorus and manganese contents by 8.2 and 8.8%. These results corroborate earlier findings that Se fertilization can modulate nutrient uptake and redistribution in cereal crops (Xue et al., 2023; Cheng et al., 2024; Shah et al., 2024). Mechanistically, Se may enhance root system architecture and membrane permeability (Zhou et al., 2023), improving nutrient acquisition and translocation efficiency (Zhou et al., 2024). At the molecular level, Se has been reported to upregulate phosphate and nitrate transporter expression, facilitating phosphorus and nitrogen assimilation (Zhou et al., 2020; Zhou et al., 2023). Se’s interaction with sulfur metabolism may further influence the mobility of divalent cations (e.g., Zn^2+^, Fe^2+^, and Mn^2+^) through competitive uptake and altered redox states (Zhou et al., 2020). The higher kernel magnesium and phosphorus contents observed under Se1 and Se2 (8.0% and 8.2% increases, respectively) may also reflect enhanced photosynthetic activity and assimilate translocation, as these nutrients play key roles in chlorophyll function, enzyme activation, and carbohydrate metabolism (Zhou et al., 2024). Overall, these responses reveal that foliar Se supply enhances both the metabolic and nutritional quality of maize kernels through coordinated effects on photosynthetic efficiency, nutrient uptake, and redox regulation. These findings underscore that optimized foliar selenium application can effectively biofortify sweet maize, enhancing its nutritional and functional quality. By increasing kernel protein, carbohydrate, and essential micronutrient contents, moderate Se supplementation may contribute to reducing hidden hunger and micronutrient deficiencies in human populations (Li et al., 2021b). However, the narrow beneficial range observed emphasizes the need for precision Se management to avoid potential toxicity and nutritional imbalances. Establishing cultivar-specific and environment-sensitive Se application thresholds is therefore crucial for translating these physiological benefits into safe, sustainable agronomic practices.

### 4.3 Effect of foliar selenium application on sweet maize selenium content, speciation, and bioaccessibility

Selenium is an essential micronutrient for human health, and its biofortification in staple crops is a promising strategy to combat Se deficiency (Shahidin et al., 2025). Foliar Se fertilization has been widely reported as an effective method for enhancing Se accumulation in crops, particularly cereals (Shah et al., 2024). When applied to the leaf surface, Se penetrates primarily through aqueous cuticular pores and epidermal uptake sites, after which it enters mesophyll cells and is mobilized through the phloem to developing tissues (Danso et al., 2023). Consistent with this mechanism, we observed a clear dose-dependent increase in Se concentrations across maize tissues (**Figures 6A–G**), in agreement with previous reports in maize and other cereals (Wang et al., 2021; Gao et al., 2024; Mrština et al., 2024). Among the various plant tissues analyzed, leaves exhibited the highest Se accumulation, followed by stem, husk, tassel, kernel, cob, and root. These results suggest that Se was efficiently absorbed and translocated to the maize tissues. Notably, Se concentrations in the kernel increased significantly following foliar application, with values reaching 0.44 mg kg^−1^, 0.64 mg kg^−1^, and 2.05 mg kg^−1^ under Se1, Se2, and Se3 treatments, respectively. This increase was substantial, with Se concentrations in the kernels rising by 86.2%, 90.4%, and 97.0% compared with the control. These results align with the findings of Deliboran et al (Deliboran, 2023), who reported similar trends in Se accumulation following foliar treatments in maize grain. The pattern of Se distribution and accumulation across maize tissues aligns with the biochemical fate of selenite in plant metabolism (Longchamp et al., 2015; Wang et al., 2021). Unlike selenate, which follows the sulfate assimilation pathway from the start, selenite bypasses sulfate transporters and instead enters via phosphate transporters and aquaporins (Wang et al., 2021). Once inside the cell, its rapid reduction integrates it downstream into the sulfur assimilation system (Somagattu et al., 2024). The selenide formed subsequently reacts with O-acetylserine through O-acetylserine(thiol)lyase, producing SeCys, which can be methylated to MeSeCys or further converted through trans-sulfuration and aminotransferase pathways to selenohomocysteine and SeMet (Trippe and Pilon-Smits, 2021). This biochemical conversion explains why organic Se species dominate in the kernels. In accordance with food safety standards set by the Standardization Administration of China (GHT1135-2017), acceptable Se concentrations in cereal grains range from 0.15 to 0.50 mg kg^−1^ (Standardization Administration of the People’s Republic of China, 2017). Thus, Se1 remained within the safe threshold for Se-enriched maize, while Se2 and Se3 exceeded the recommended upper limit. This highlights the importance of optimizing application rates to balance nutritional value with safety.

The nutritional quality of Se-biofortified crops depends not only on total Se content but also on the chemical forms present, since different Se species exhibit varying degrees of bioavailability and toxicity (Tangjaidee et al., 2023). In plants, Se exists in both inorganic forms, including selenite (Se^4+^) and selenate (Se^6+^), as well as organic compounds such as selenomethionine (SeMet), selenocysteine (SeCys), and methylselenocysteine (MeSeCys) (Luo et al., 2025). The organic forms are generally regarded as more bioavailable and less toxic (Muleya et al., 2021). In this study, SeMet accounted for the majority of Se in sweet maize kernels (82.3%), followed by SeCys (16.0%) and MeSeCys (9.3%) under Se1 treatment (**Figure 7**), consistent with previous observations in cereals (Lu et al., 2018). The predominance of organic Se species suggests that foliar Se application may favor enzymatic conversion of inorganic Se into bioavailable forms, potentially through interactions with sulfur-assimilation pathways (Gong et al., 2018). From a nutritional standpoint, SeMet is particularly valuable due to its high gastrointestinal bioavailability and its capacity for nonspecific incorporation into body proteins via methionine substitution, thereby acting as a long-term Se reservoir (Fairweather-Tait et al., 2010). Since previous studies have reported bioavailability exceeding 90% for SeMet-rich plant foods (Fairweather-Tait et al., 2010), the Se-biofortified sweet maize obtained under moderate Se application conditions could represent an effective dietary Se source in regions with insufficient Se intake. The predominance of organic Se species observed in the current study thus supports the potential of foliar biofortification as a viable approach for producing nutritionally enhanced maize while minimizing the risks associated with inorganic Se accumulation.

Selenium bioaccessibility, the fraction of total Se released from the food matrix during digestion, provides further insight into the nutritional efficiency of Se-biofortified crops. In this study, Se bioaccessibility was significantly higher in the gastrointestinal (GI) phase (37.67–76.00%) than in the gastric (G) phase (14.67–35.60%), reflecting distinct Se release dynamics across digestion stages. Increasing foliar Se application rates corresponded with higher bioaccessibility in both phases, suggesting a dose-dependent enhancement of Se release. These findings align with previous reports (Muleya et al., 2021; Zeng et al., 2023) indicating that digestion conditions play a major role in Se liberation from maize matrices. The relatively low Se bioaccessibility observed in the gastric phase may be associated with the formation of stable Se–protein and Se-carbohydrate complexes that resist hydrolysis under acidic conditions (Pyrzynska and Sentkowska, 2021; Abdalla et al., 2025). Conversely, the increased bioaccessibility observed during the GI phase may be attributed to optimal pH conditions, enzymatic hydrolysis, and bile salt activity, which facilitated the breakdown of these complexes. The high proportions of organic Se species (particularly SeMet) likely contribute to the observed bioaccessibility, as these forms tend to be more soluble and readily absorbed in the small intestine (Muleya et al., 2021). Similar results have been reported by Zeng et al (Zeng et al., 2023), who recorded GI-phase Se bioaccessibility of 82% compared with 28% in the G phase. Studies conducted in Malawi also reported that Se-biofortified maize containing approximately 90% SeMet achieved approximately 74% GI-phase bioaccessibility (Muleya et al., 2021), further emphasizing the importance of Se speciation for nutritional outcomes. Overall, the combined evidence from Se accumulation, speciation, and bioaccessibility analyses in the present study demonstrates that moderate foliar Se application enhances the nutritional quality of sweet maize by increasing both total Se concentration and the proportion of bioavailable organic Se forms. At the same time, excessive Se application may lead to concentrations exceeding food safety limits. These findings underscore the value of defining optimal Se application thresholds that maximize the nutritional efficacy of Se biofortification while ensuring consumer safety. This integrative evaluation provides a basis for developing targeted Se fertilization strategies that enhance dietary Se intake through maize-based food systems.

### 4.4 Mechanisms of selenium metabolic transport and accumulation

**Figure 8:**
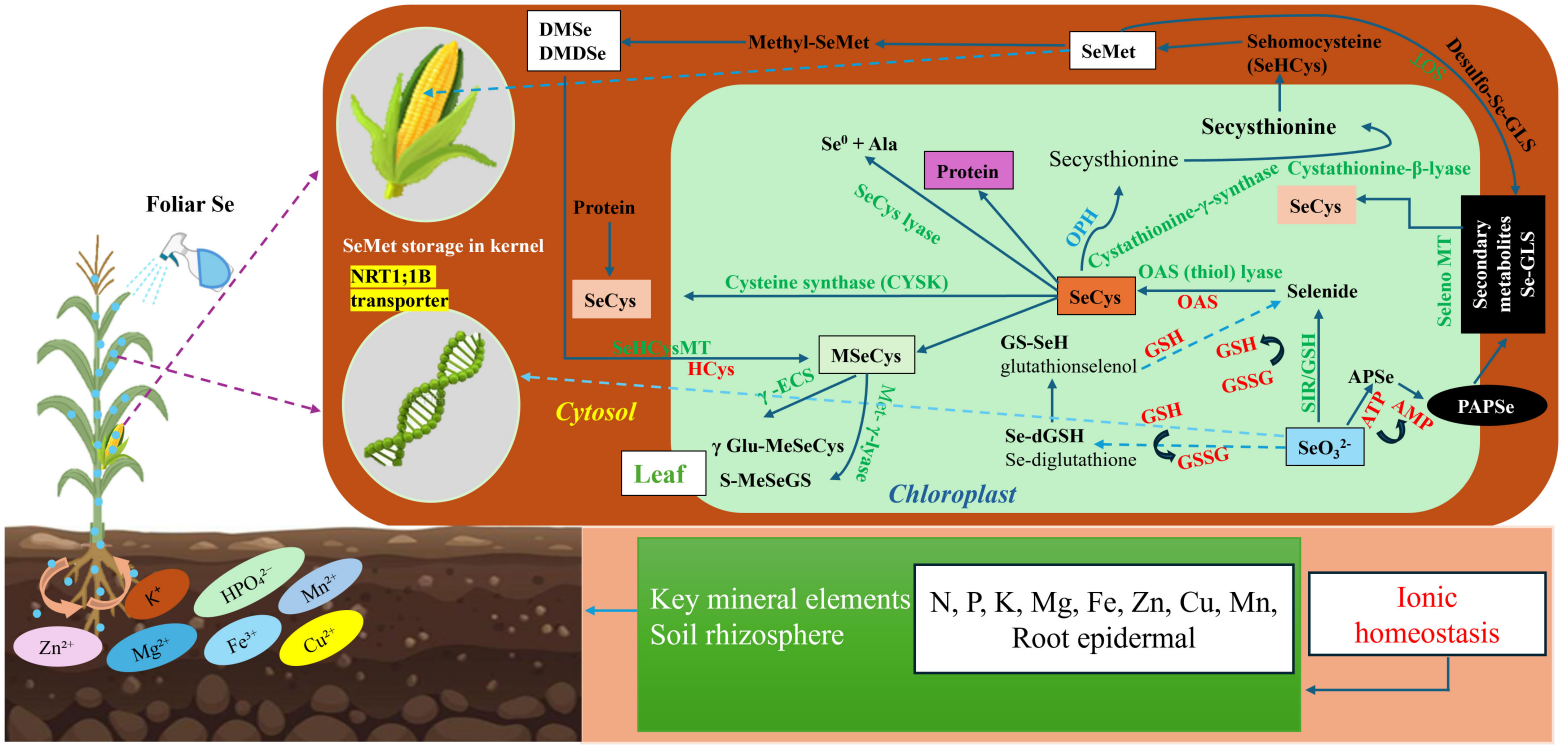
Schematic illustration of selenium (Se) uptake, translocation, and metabolism in sweet maize kernels. Foliar Se absorption occurs primarily through leaf epidermal and cuticular pathways, involving transporters such as NRT1;1. Enzymes shown in red indicate potential regulatory activity mediated by adenosine 5’-phosphosulfate (APS). Enzyme and metabolite abbreviations: TRXR, thioredoxin reductase; CS, cysteine synthase; SiR, sulfite reductase; CYSK, cysteine synthase; GSH, glutathione; GSSG, glutathione disulfide; OPH, O-phosphohomoserine; CGS, cystathionine γ-synthase; SMT, selenocysteine methyltransferase; MMT, methionine S-methyltransferase; MS, methionine synthase; GR, glutathione reductase; Hcy, homocysteine; SOT, seleno-organic transferase; Ala, alanine.

Foliar application of selenite (SeO_3_^2−^) in maize triggers a distinct metabolic pathway that bypasses the conventional soil–root uptake process and directly engages the leaf’s biochemical systems (**Figure 8**). Upon deposition on the leaf surface, SeO_3_^2−^ penetrates the cuticular layer, primarily through aqueous pores, with partial entry occurring via stomatal openings (Yang et al., 2019). Surfactants further facilitate this process by reducing cuticular resistance, which increases uptake efficiency (Somagattu et al., 2024). Once within the apoplast, SeO_3_^2−^ is absorbed by mesophyll cells via phosphate transporters, which exhibit substrate promiscuity, allowing SeO_3_^2−^ to mimic phosphate and thereby enter the plant’s intracellular environment (Yang et al., 2019; Wang et al., 2021). Inside the cytosol, SeO_3_^2−^ encounters the highly reducing conditions of the leaf, where it undergoes a series of biochemical transformations. Through a glutathione-dependent reduction pathway, SeO_3_^2−^ is first converted into selenodiglutathione (GS–Se–SG), and subsequently reduced to selenide (Se^2−^) (Yang et al., 2019). Glutaredoxins play a critical role in maintaining the redox balance during this transition (Sevilla et al., 2023). This reduction process serves as a detoxification checkpoint, ensuring that Se is safely channeled into the plant’s sulfur assimilation pathway, which is particularly active in photosynthetic tissues (Somagattu et al., 2024). The produced Se^2−^ is then incorporated into the O-acetylserine pool via the action of O-acetylserine(thiol)lyase (OASTL), leading to the formation of selenocysteine (SeCys), the first stable organic form of Se in the plant (Yang et al., 2019). Selenocysteine, however, is chemically reactive and poses potential risks to cellular integrity by disrupting protein structure (Kolbert et al., 2019). To mitigate this, maize leaves redirect SeCys through two distinct metabolic pathways. The first pathway involves trans-sulfuration and methionine biosynthesis, which results in the production of selenomethionine (SeMet) (Zhang et al., 2020). SeMet is structurally similar to methionine, allowing it to integrate seamlessly into the general amino acid pool, and it can be incorporated into proteins during translation (Zhang et al., 2020). The second pathway involves the methylation of SeCys to form methylselenocysteine (MeSeCys), a less reactive and more stable form of Se that serves as a storage compound (Li J. et al., 2025). The regulation of these conversion pathways is influenced by several factors, including the availability of thiols, the cellular redox status, and the metabolic demand for sulfur analogs (Zhou et al., 2024). This delicate balance between detoxification and nutrient assimilation ensures that Se is both safely managed within the plant and incorporated into metabolic processes that are beneficial to growth and development (Gupta and Gupta, 2017; Zhou et al., 2024). In contrast to root-fed selenate, which depends on xylem transport, organic Se forms derived from foliar applications are efficiently loaded into the phloem (Gupta and Gupta, 2017). This transport is facilitated by amino acid permeases and lysine-histidine-type transporters, which assist in the mobilization of selenium along with other assimilates (Zhou et al., 2024). As a result, Se is redistributed from the treated leaves to other plant tissues, including leaves, stems, roots, and reproductive organs, particularly during grain filling. The speciation of Se in maize kernels is predominantly governed by metabolic processes occurring in the source leaves. SeMet is the primary form found in the grain due to its structural similarity to methionine, which facilitates its incorporation into storage proteins and efficient transport via the phloem (Abdalla et al., 2025). Although small amounts of SeCys may be present, it is typically converted to SeMet or methylated in developing kernels. The final Se profile in maize grain reflects the combined effects of foliar assimilation, phloem transport, and kernel biosynthesis, demonstrating the maize plant’s adaptive strategies for Se management.

### 4.5 Limitations of the study

While this study demonstrates the benefits of foliar Se application, it is important to recognize that the findings are based on a single growing season, location, and sweet maize cultivar. Consequently, the broader applicability of these results may be influenced by variations in environmental conditions, soil properties, and genetic factors, all of which can affect plant responses to Se. Therefore, although moderate Se application appears to enhance physiological responses, the extent of agronomic benefits may differ under varying conditions. To further evaluate the robustness and sustainability of foliar Se application, long-term, multi-site studies across diverse growing seasons and genetic backgrounds are essential. Future research should also investigate a wider range of cultivars and agroecological contexts to assess the stability of Se-mediated responses in different field environments. Additionally, incorporating transcriptomic and metabolomic approaches would provide deeper insights into the molecular mechanisms of Se uptake, transport, assimilation, and its interactions with redox and nutrient regulation.

## 5 Conclusion

This study demonstrates that foliar Se application exerts a clear dose-dependent influence on the physiological, biochemical, and nutritional attributes of sweet maize. Moderate Se doses (20–40 g ha^−1^) were associated with improved photosynthetic efficiency, elevated antioxidant enzyme activity (superoxide dismutase, peroxidase, catalase), and enhanced carbohydrate metabolism, collectively contributing to higher kernel yield and improved nutritional quality. In contrast, the highest application rate (60 g ha^−1^) appeared to disrupt redox balance, constrain nutrient assimilation, and diminish grain quality. These findings support the interpretation that Se functions as both a beneficial micronutrient and a modulator of redox homeostasis within a narrow optimal range. At moderate levels, foliar Se application was associated with enhanced antioxidant defense systems, possibly through the activation of enzymes such as glutathione peroxidase, thioredoxin reductase, and catalase, thus helping to maintain chloroplast stability and support the translocation of assimilates. Se supplementation also appeared to influence nutrient transport processes, possibly by affecting membrane permeability and regulating sulfate and phosphate transporters, thus improving mineral uptake and redistribution. Furthermore, foliar Se fertilization increased kernel Se concentration and favored the conversion of inorganic Se into nutritionally desirable organic forms, predominantly selenomethionine (SeMet), which accounted for over 80% of total Se. Overall, these findings position moderate foliar Se application as a promising biofortification strategy, particularly for regions facing dietary Se insufficiency. The study thus provides a substantive foundation for future investigations aimed at refining and scaling Se-based agronomic interventions for improved crop performance and human nutrition.

## Data Availability

The original contributions presented in the study are included in the article/**Supplementary Material**. Further inquiries can be directed to the corresponding author/s.
