# Supplementary material for: Foliar application of selenium increased selenium accumulation, speciation, and bioaccessibility, as well as the yield and nutritional quality of sweet maize
Source: Front Plant Sci. 2025 Dec 19;16:1733890. doi: 10.3389/fpls.2025.1733890 (PMC12757374; doi:10.3389/fpls.2025.1733890)
Supplement: Supplementary file 1 [file Table1.docx]

Supplementary Material

# Supplementary Text

## Text S1. Determination of Selenium content in sweet maize organs

The total Se contents in the grounded maize organ samples, including kernel, leaf, stem, root, cob, husk, and tassel, were quantified using an inductively coupled plasma mass spectrometry (ICP-MS, 7900, Agilent, Santa Clara, CA, USA) approach adapted with specific modifications. To ensure analytical reliability, all samples were prepared and analyzed in triplicate. Precisely, 0.8 g of each ground maize organ part was placed into a microwave digestion vessel, to which 10 mL of nitric acid was added. The mixture was allowed to soak for 12 hours. Subsequently, 2.5 mL of 30% hydrogen peroxide was added and allowed to react for 40 minutes. The digestion tube was then transferred to a microwave for the digestion process**.** Upon completion of digestion, the tubes were removed and allowed to cool to room temperature. The samples were then placed in a heating furnace to evaporate the residual acid until approximately 1.5 mL of residue remained. After cooling to room temperature, the concentrated solution was carefully transferred into a 25 mL volumetric flask and diluted to the mark with deionized water. The Se content in each organ was subsequently quantified using ICP-MS.

## Text S2. Determination of Selenium speciation in sweet maize kernels

The characterization and quantification of Se species in sweet maize kernels were conducted using liquid chromatography coupled with inductively coupled plasma mass spectrometry (LC-ICP-MS). Specifically, approximately 0.8 g of finely ground kernel flour was weighed into a centrifuge tube. Six mL of 60 mg/L Pronase E solution was added to the sample. The mixture underwent ultrasonication at 38 °C for 35 minutes to facilitate the enzymatic hydrolysis of Se-containing compounds. The resulting suspension was centrifuged at 10,000 × g for 25 minutes at 4 °C to isolate the supernatant, which contained the extracted Se species. The supernatant was carefully decanted and filtered through a 0.22 μm syringe filter to remove any remaining residual particulates. The filtered extract was promptly stored at -80 °C in airtight vials to prevent Se species degradation until LC-ICP-MS analysis. Chromatographic separation was performed using a Hamilton PRP-X100 reversed-phase anion exchange column (4.0 × 250 mm, 10 μm)**.** The mobile phase consisted of ammonium hydrogen phosphate ((NH₄)₂HPO₄) solutions at 35 mmol L⁻¹ (mobile phase X) and 75 mmol L⁻¹ (mobile phase Y), delivered at a flow rate of 1.5 mL min⁻¹ under a gradient elution protocol specifically optimized for resolving Se species, including SeMet, SeCys, and MeSeCys. Calibration curves were established using certified standards of each Se species, and quality control samples were analyzed to verify method accuracy and precision, as presented in **Table S3.**

## Text S3. Quantification of soluble sugar content in sweet maize kernels

The concentration of soluble sugars in sweet maize kernels was determined using a modified anthrone colorimetry. Initially, 0.5 g of kernel flour was weighed and placed into a suitable container. Soluble sugars were extracted by adding 5 mL of 80% ethanol, followed by thorough mixing to achieve a homogeneous suspension. The mixture was incubated in a water bath at 85°C for 40 minutes to optimize sugar extraction. Upon cooling to ambient temperature, the suspension was centrifuged at 3200 rpm for 20 minutes to isolate the supernatant. This extraction process was repeated three times, and the collected supernatants were pooled. To eliminate potential interfering substances, the combined supernatant was filtered through activated carbon, and the volume was adjusted to 50 mL with distilled water to prepare the test solution. For the colorimetric analysis, 1.5 mL of the test solution was mixed with 1.5 mL of distilled water and 4.5 mL of 0.2% anthrone reagent in a sealed test tube. The mixture was vigorously mixed and heated in a boiling water bath for 20 minutes to induce the characteristic colour development. After cooling to 25°C, the optical density was measured at a wavelength of 620 nm using a UV-visible spectrophotometer. The soluble sugar concentration was determined by comparing against a calibration curve established with glucose standards ranging from 0 to 100 mg/L, as detailed in **Table S1**.

## Text S4. Analysis of crude fat content in sweet maize kernels

A modified Soxhlet extraction method was used to determine crude fat content in sweet maize. Precisely 3.0 g of maize flour was weighed and transferred into a filter paper tube. This tube was positioned in the extraction chamber of a Soxhlet extractor, connected to a pre-dried receiving flask, pre-weighed to a constant mass. Light petroleum was added to the receiving flask, filling it to about two-thirds of its capacity. Extraction was conducted by heating the flask in a water bath, enabling continuous reflux of the light petroleum for 9 hours. The receiving flask was evaporated until approximately 1–2 mL of solvent remained. The residual solvent was removed using a water bath, followed by drying the flask in an oven at 105 °C for 1.5 hours. After cooling in a desiccator for 40 minutes, the flask was weighed. The crude fat content, expressed as a percentage, was computed using the formula:

Crude fat content (%) = n1 – n0 / n2 x 100 (3)

Where n0 is the mass of the pre-dried receiving flask, n1 is the mass of the flask with extracted fat, and n2 is the mass of the maize flour sample (3.0 g).

## Text S5. Determination of vitamin C content in sweet maize kernels

The molybdenum blue colorimetric method was employed to determine vitamin C content in sweet maize kernels with specific modifications. Briefly, a 0.5 g sample of sweet maize flour was weighed and placed into a 25 mL volumetric flask. The sample was dissolved by adding an oxalic acid–EDTA solution (0.6% oxalic acid, 0.15% EDTA) to preserve ascorbic acid stability, with the volume adjusted to 25 mL using the same solution. The mixture was vigorously mixed to ensure homogeneity and then centrifuged at 3200 rpm for 12 minutes to eliminate insoluble materials. A 12 mL aliquot of the resulting supernatant was transferred to a separate container. Subsequently, 1.2 mL of 5% metaphosphoric acid–10% acetic acid solution was added to enhance ascorbic acid stability, followed by 2.5 mL of 5% sulfuric acid to ensure an acidic medium, and 4.5 mL of 5% ammonium molybdate to initiate the colorimetric reaction. The solution was diluted to a final volume of 25 mL with distilled water and mixed thoroughly. The mixture was incubated at ambient temperature for 18 minutes to facilitate the development of the phosphomolybdenum blue complex. Optical density was then measured at a wavelength of 705 nm using a UV-visible spectrophotometer. The ascorbic acid concentration was determined using a tailored standard calibration curve, as shown in **Table S1**.

## Text S6. Quantification of trace element content in sweet maize kernels

The determination of trace element content (N, P, K, Mg, Mn, Fe, Cu, Zn) in sweet maize kernels was conducted using an adapted inductively coupled plasma optical emission spectrometry (ICP-OES) method with specific modifications. A 0.8 g sample of kernel flour was precisely weighed and transferred into a polytetrafluoroethylene digestion vessel. To this, 10 mL of 65% nitric acid and 3 mL of 30% hydrogen peroxide were added. Digestion was performed using a BerghofXpert microwave digestion system, employing a refined four-step temperature protocol: 150 °C, 175 °C, 195 °C, and a final cooling phase at 55 °C, totaling 45 minutes. Post-digestion, the vessels were allowed to cool to room temperature, and the digest was diluted to a consistent volume with ultrapure water. The resulting solution was analyzed for P, K, Mg, Mn, Fe, Cu, and Zn concentrations using an ICP-OES system (Perkin Elmer Optima 5300DV). Calibration standards for each element were prepared and measured to ensure precise quantification. Blank samples, processed with identical reagents excluding maize powder, were included in each batch for quality assurance, as detailed in **Table S2**. Nitrogen content was assessed using the Kjeldahl method, as outlined for crude protein quantification.

## Text S7. Determination of crude protein content in sweet maize kernels

The Kjeldahl method was employed with slight modifications to quantify crude protein content in sweet maize kernels. A 0.8 g sample of sweet maize flour was accurately weighed and placed into a digestion flask of the K9840 Kjeldahl Analyzer. Concentrated sulfuric acid was added, and the mixture was heated to 385 °C for 100 minutes to facilitate digestion. A Kjeldahl catalyst was incorporated to enhance the complete breakdown and oxidation of the sample, converting organic nitrogen into ammonium sulfate. Digestion proceeded until the solution achieved clarity, signifying thorough decomposition. After cooling, sodium hydroxide solution was introduced to release ammonia gas. The ammonia was distilled using the K9840 Kjeldahl Analyzer, collected in a receiving flask containing a standard acid solution with an indicator. Ammonia content was determined via back titration, where excess acid in the receiving flask was neutralized with a standardized sodium hydroxide solution to ascertain the absorbed ammonia quantity. Total nitrogen content was calculated based on the volume of sodium hydroxide used in the titration. Crude protein content was subsequently derived by applying a conversion factor of 6.25 to the nitrogen value.

## Text S8. Analysis of total starch in sweet maize kernels

The total starch content was quantified using a modified anthrone colorimetry method. Sweet maize flour samples were defatted using petroleum ether to remove lipids. A precise 1.0 g portion of the defatted flour was weighed and subjected to an adapted extraction process, similar to that used for soluble sugars, to separate the supernatant and precipitate. The supernatant was discarded, retaining only the starch-rich precipitate for subsequent steps. This precipitate was placed into a 100 mL conical flask, where 12 mL of 6 mol/L hydrochloric acid was introduced. The mixture was heated in a boiling water bath for 15 minutes, with the progress of hydrolysis tracked by testing small samples with an iodine reagent until no blue colour was formed, confirming complete starch breakdown. After cooling to room temperature, 25 mL of distilled water was added and mixed thoroughly. The hydrolyzed solution was filtered into a 50 mL volumetric flask, with the volume brought to 50 mL using distilled water. From this, 1.5 mL was transferred to a stoppered test tube, and 5 mL of anhydrous ethanol was added, followed by vigorous shaking to ensure uniformity. Then, 1 mL of this ethanol-treated sample was taken for analysis. A tailored anthrone colorimetric approach was employed, adding 0.2% anthrone in concentrated sulfuric acid, and the mixture was heated to produce a blue-green hue. The optical density was recorded at a wavelength of 620 nm using a spectrophotometer. Starch content was determined by comparing absorbance values to a custom standard curve developed with known glucose standards, adjusted with a starch-to-glucose conversion factor derived from **Table 1.**

## Text S9. Analysis of apparent amylose content in sweet maize kernels

A modified dual-wavelength iodine-binding spectrophotometric method was employed to quantify amylose content in maize kernels. An initial 0.2 g aliquot of previously defatted maize flour was accurately weighed and transferred to a beaker. The mixture was heated in a boiling water bath for 12 minutes to ensure complete starch dissolution. Following cooling to room temperature, the solution was transferred to a 50 mL volumetric flask, with the volume adjusted to 50 mL using distilled water, and allowed to stand for 20 minutes. The mixture was subsequently filtered to remove insoluble residues. Six mL of the filtrate were combined with 30 mL of distilled water in a separate container. The pH was adjusted to approximately 3.6 using 0.1 mol/L hydrochloric acid, as confirmed by a pH meter. Then, 0.6 mL of iodine reagent was added, and the solution was diluted to 50 mL with distilled water, followed by thorough mixing. After a 30-minute incubation period to facilitate complete colour development of the amylose-iodine complex, absorbance was measured at dual wavelengths (620 nm and 510 nm) using a spectrophotometer, with a blank control prepared from distilled water. Amylose content was determined using a custom standard curve constructed from known amylose concentrations, as outlined in **Table S1**.

## Text S10. Determination of Selenium bioaccessibility in sweet maize kernels

The bioaccessible Se fraction in sweet maize kernels was assessed using a Modified Physiologically Based Extraction Test (MPBET). Initially, a 2.5 g sample of sweet maize kernel flour was accurately weighed and placed into a 50 mL centrifuge tube. 25 mL of gastric phase fluid, composed of 0.60 g/L citric acid, 0.60 g/L maleic acid, 1.50 g/L pepsin, 450 μL/L lactic acid, and 550 μL/L acetic acid in Milli-Q water, pre-adjusted to pH 2.6 with HCl solution, was added to the tube. The mixture was agitated at 160 rpm for 75 minutes at 37 °C, with the pH maintained at 2.6 by readjusting every 25 minutes using HCl solution. A 2.5 mL aliquot of the supernatant was collected and filtered through a 0.45 μm membrane filter. The filtered extract was stored at 4 °C pending analysis. The pH of the remaining gastric phase mixture was adjusted to 7.5 using a saturated solution of sodium bicarbonate. Two and a half mL of intestinal phase fluid, containing 0.6 g/L pancreatin and 2.0 g/L bile in Milli-Q water, were added to the mixture. The solution was oscillated at 160 rpm for 4.5 hours at 37 °C, with the pH held at 7.5 by adjusting it every 2.5 hours with sodium bicarbonate solution. The mixture was then centrifuged at 4000 × g for 15 minutes, and the supernatant was filtered through a 0.45 μm membrane filter. The filtered extract was stored at 4 °C until analysis. The filtered extracts from both the gastric (G) and intestinal (GI) phases were analyzed for Se content using ICP-MS, employing external calibration standards before measurements. Selenium bioaccessibility (BA%) was then calculated using the equation:

$\mathrm{BA}\left( \% \right)=\frac{Se in G or GI}{Total Se in sample}X 100\%$ (2)

where Se in G or GI represents the Se content (mg/kg) in the gastric or gastrointestinal phases, and Total Se in the sample denotes the Se content in the corresponding kernel samples.

## Text S11. Assessment of the activity of SOD, POD, and CAT in fresh sweet maize leaf

Fresh maize leaf tissues were rapidly frozen in liquid nitrogen (N₂) and ground into a fine powder to prepare a homogenate. The homogenate was subsequently mixed with 8.5 ml of 50 mM sodium phosphate buffer (pH 7.8) and centrifuged at 12,000 g (8000 rpm) for 15 minutes at 4.5 °C to remove cellular debris. The resulting supernatant was used for enzyme assays.

Superoxide Dismutase (SOD) Activity

SOD activity was measured by assessing the inhibition of nitro blue tetrazolium (NBT) reduction at 560 nm. The assay was conducted at pH 7.8, and one unit of activity was defined as the amount of enzyme that causes a 50% reduction in NBT reduction rate.

Peroxidase (POD) Activity

The POD activity assay mixture contained 0.5 mL of sodium phosphate buffer (pH 7.5), 0.95 mL of 0.1% (w/v) guaiacol solution, 0.5 mL of 0.2% (v/v) hydrogen peroxide, and 0.05 mL of enzyme extract. The reaction was initiated by the addition of H₂O₂, and the increase in absorbance at 470 nm was recorded for 90 seconds, with measurements taken every 30 seconds. POD activity was quantified as the amount of enzyme required to oxidize 0.5 mg of guaiacol at 470 nm.

Catalase (CAT) Activity

CAT activity was measured by mixing 1.85 mL of deionized water with 0.5 mL of 0.2% (v/v) H₂O₂ and 0.05 mL of enzyme extract. The breakdown of H₂O₂ was monitored by measuring the decrease in absorbance at 240 nm over a 90-second period, with readings taken every 30 seconds. One unit of CAT activity was defined as the amount of enzyme required to degrade 0.5 μmol of H₂O₂ per minute per gram of fresh leaf tissue. The pH for CAT assays was set to 7.0.

Text S12 Assessment of MDA content in fresh sweet maize leaf

Fresh maize leaf samples (1.0 g) were homogenized in 12% trichloroacetic acid and centrifuged to obtain the supernatant. The resulting supernatant (2.5 ml) was then mixed with 2.5 ml of 0.7% thiobarbituric acid (TBA) solution and incubated in a boiling water bath for 15 minutes. The absorbance of the reaction mixture was recorded at 450, 532, and 600 nm using a microplate reader.

# Supplementary Tables

## Table S1. Calibration Curve equations for determining the Nutritional Composition

| **Analyte Calibration Equation Concentration Range R^2^** |
| --- |

Soluble sugar *y* = 181.9*x* - 0.504 0 mg / L - 100 mg / L 0.997

Vitamin C *y* = 4.8*x* + 0.009 0 mg / L - 1.4 mg / L 0.999

Total starch *y* = 2.4*x* + 0.068 0 mg / L - 100 mg / L 0.999

Amylose *y* = 5.3*x* − 0.025 0 mg / L - 2 mg / L 0.999

## Analytical Spectra for the Mineral Elements Measured

| **Mineral elements Analyze spectral line wavelength (nm)** |
| --- |

P 213.60

K 766.49

Mg 279.08

Zn 206.20

Cu 324.75

Fe 239.50

Mn 257.60

## Table S3. Calibration Parameters for Selenium Species Determined by LC-ICP-MS

| **Se speciation Standard curve R^2^** |
| --- |

SeMet *y* = 4527.14**x* + 1137.62 0.9992

SeCys *y* = 8028.22**x* + 479.67 0.9999

MeSeCys *y* = 6991.54**x* + 417.96 0.9999
